# Genetic consequences of effective and suboptimal dosing with mutagenic drugs in a hamster model of SARS-CoV-2 infection

**DOI:** 10.1093/ve/veae001

**Published:** 2024-01-04

**Authors:** Christopher J. R Illingworth, Jose A Guerra-Assuncao, Samuel Gregg, Oscar Charles, Juanita Pang, Sunando Roy, Rana Abdelnabi, Johan Neyts, Judith Breuer

**Affiliations:** MRC-University of Glasgow Centre for Virus Research, 464 Bearsden Road, Glasgow G61 1QH, UK; Great Ormond Street Hospital for Children NHS Foundation Trust, Great Ormond Street, London WC1N 3JH, UK; Infection, Immunity and Inflammation Research and Teaching Department, University College London, Gower Street, London WC1E 6BT, UK; Infection, Immunity and Inflammation Research and Teaching Department, University College London, Gower Street, London WC1E 6BT, UK; Great Ormond Street Hospital for Children NHS Foundation Trust, Great Ormond Street, London WC1N 3JH, UK; Infection, Immunity and Inflammation Research and Teaching Department, University College London, Gower Street, London WC1E 6BT, UK; Infection, Immunity and Inflammation Research and Teaching Department, University College London, Gower Street, London WC1E 6BT, UK; Infection, Immunity and Inflammation Research and Teaching Department, University College London, Gower Street, London WC1E 6BT, UK; KU Leuven Department of Microbiology, Immunology and Transplantation, Rega Institute for Medical Research, Laboratory of Virology and Chemotherapy, Herestraat 49, Leuven B-3000, Belgium; The VirusBank Platform, Gaston Geenslaan, Leuven B-3000, Belgium; KU Leuven Department of Microbiology, Immunology and Transplantation, Rega Institute for Medical Research, Laboratory of Virology and Chemotherapy, Herestraat 49, Leuven B-3000, Belgium; The VirusBank Platform, Gaston Geenslaan, Leuven B-3000, Belgium; Great Ormond Street Hospital for Children NHS Foundation Trust, Great Ormond Street, London WC1N 3JH, UK; Infection, Immunity and Inflammation Research and Teaching Department, University College London, Gower Street, London WC1E 6BT, UK

**Keywords:** SARS-CoV-2, favipiravir, molnupiravir, mutagenesis

## Abstract

Mutagenic antiviral drugs have shown promise against multiple viruses, but concerns have been raised about whether their use might promote the emergence of new and harmful viral variants. Recently, genetic signatures associated with molnupiravir use have been identified in the global SARS-COV-2 population. Here, we examine the consequences of using favipiravir and molnupiravir to treat SARS-CoV-2 infection in a hamster model, comparing viral genome sequence data collected from (1) untreated hamsters, and (2) from hamsters receiving effective and suboptimal doses of treatment. We identify a broadly linear relationship between drug dose and the extent of variation in treated viral populations, with a high proportion of this variation being composed of variants at frequencies of less than 1 per cent, below typical thresholds for variant calling. Treatment with an effective dose of antiviral drug was associated with a gain of between 7 and 10 variants per viral genome relative to drug-free controls: even after a short period of treatment a population founded by a transmitted virus could contain multiple sequence differences to that of the original host. Treatment with a suboptimal dose of drug showed intermediate gains of variants. No dose-dependent signal was identified in the numbers of single-nucleotide variants reaching frequencies in excess of 5 per cent. We did not find evidence to support the emergence of drug resistance or of novel immune phenotypes. Our study suggests that where onward transmission occurs, a short period of treatment with mutagenic drugs may be sufficient to generate a significant increase in the number of viral variants transmitted.

## Introduction

Molnupiravir (EIDD-2801/MK-4482) is an oral prodrug of the nucleoside analogue β-D-N4-hydroxycytidine (EIDD-1931, NHC) with broad inhibitory activity against RNA-dependent RNA polymerases of positive- and negative-strand RNA viruses (RdRps) including SARS-CoV-2 and other coronaviruses (Reynard et al. 2015; Yoon et al. 2018; Agostini et al. 2019; Painter et al. 2019; Kabinger et al. 2021; Wahl et al. 2021). Following oral administration, molnupiravir is metabolized and phosphorylated into the active drug NHC 5ʹ-triphosphate (NHC-TP). During viral replication, NHC-TP is substituted by SARS-CoV-2 RdRp for cytidine or uridine triphosphate into newly synthesized viral RNA, resulting in incorporation of either guanosine or adenosine when the RNA is copied (Kabinger et al. 2021; Malone and Campbell 2021). The accumulation of random C to U or G to A transition errors in the viral RNA genome is associated with lethal mutagenesis resulting in loss of viral fitness as evidenced by reduced viral loads, reduced viral infectivity, and reduced lung pathology in animal models (Sheahan et al. 2020; Fischer et al. 2022). Favipiravir, a nucleoside analogue, has also been associated with C to U and G to A transition errors and lethal mutagenesis (Lumby et al. 2020; Sheahan et al. 2020). A meta-analysis of small clinical trials suggested that favipiravir led to a statistically significant improvement over 7 days in hospitalised SARS-CoV-2 patients as measured on the WHO Ordinal Scale (Marshall et al. 2020), although the data collected to the date of that study were insufficient to show a significant reduction in viral load or in rates of mortality or transfer to intensive care units (Hassanipour et al. 2021).

There is a substantial history of theoretical work examining the robustness of viruses to mutation and the potential for mutagenic drugs to treat viral infection (Lynch et al. 1993; Lynch, Conery, and Burger 1995; Bull, Sanjuán, and Wilke 2007; Lauring, Frydman, and Andino 2013; Stern et al. 2014; Matuszewski et al. 2017; Pénisson et al. 2017; Jensen et al. 2020). On the basis of evidence gathered from other viruses, and theoretical insights, the potential use of mutagenic antiviral drugs was suggested early in the SARS-CoV-2 pandemic (Jensen and Lynch 2020). When administered in a randomised phase 3 clinical trial to SARS-CoV-2-positive individuals who were symptomatic for 5 days or fewer, molnupiravir taken for 5 days was reported to reduce rates of hospitalisation and death (Jayk Bernal et al. 2022). Against a background of concerns about efficacy, it has been licensed by the Food and Drug Administration (FDA) for use in mild to moderate coronavirus disease (COVID-19) where the person was at increased risk for progression to severe disease, and under similar conditions for use in the UK (Dyer 2021; Mahase 2021; Thorlund et al. 2022). A recent clinical trial has shown that molnupiravir increased the transition:transversion mutation ratio in SARS-CoV-2 (Donovan-Banfield et al. 2022). The potential use of molnupiravir in combination with favipiravir has also been suggested (Eloy et al. 2021). However, concerns have been raised that the mutagenic action of molnupiravir could drive the emergence of drug-resistance mutations against itself or other antiviral therapies (Singh et al. 2021; Swanstrom and Schinazi 2022), while a recent manuscript has suggested that molnupiravir usage may have left a mutational signature upon the global SARS-CoV-2 viral population (Sanderson et al. 2023). Particular concerns have been raised about the use of sublethal doses of mutagenic drugs, whereby treatment induces viral mutagenesis without significantly reducing viral load (Nelson and Otto 2021). In this context, there is a need for a better understanding of how the use of mutagenic drugs can affect viral populations.

A combination of evolutionary experimentation and genome sequencing has been applied in various contexts to study antiviral mutagenesis. We previously used a Syrian hamster model to demonstrate the efficacy of short-term (4 days) molnupiravir treatment on reduction of SARS-CoV-2 viral load, infectivity, and lung pathology (Abdelnabi et al. 2021a). In this model, hamsters were infected with an intranasal inoculum of 50 μl, containing 2 × 10^6^ TCID_50_ SARS-CoV-2 (day 0). Treatment was initiated within an hour of the infection being founded, with animals being treated with either favipiravir, molnupiravir, a combination of the two drugs, or no drug treatment. A dose of 200 mg/kg BID (twice per day) of molnupiravir was shown to be effective in reducing infectious virus in the lung by 1.8–2.0 log_10_ TCID_50_/mg (median Tissue Culture Infectious Dose) lung tissue, depending on the SARS-CoV-2 variant used for challenge, with evidence of significantly reduced lung pathology in treated animals. In similar experiments, treatment with 300 mg/kg BID of favipiravir for 4 consecutive days achieved a reduction of nearly 2 log_10_ TCID_50_/mg in infectious virus titres in the lungs. On day 4, animals were euthanised for sampling of viral material from the lungs; during the intervening time the viral population may have undergone around 8–10 cycles of replication (Bar-On et al. 2020). Data describing viral loads are reported in the original experimental papers (Kaptein et al. 2020; Abdelnabi et al. 2021a,b). The short period of replication under drug treatment mirrors the use of molnupiravir in humans, with the advantage of the increased replicability afforded by an animal study.

Here, using viral sequence data from these hamster studies, we examine the impact of effective and suboptimal dosing with molnupiravir (200 mg/kg BID and <200 mg/kg BID, respectively) (Abdelnabi et al. 2021b) and favipiravir (300 mg/kg BID and <300 mg/kg BID, respectively) (Kaptein et al. 2020) upon viral genetic diversity. We assess different metrics for evaluating sequence diversity in treated and untreated populations. By comparing data from animals treated with different mutagenic drugs, we consider the potential for sequence-based statistics to be used as a biomarker for clinical efficacy. We discuss the potential influence of mutagenic drugs upon a transmitted viral population.

## Results

**Figure 1.**
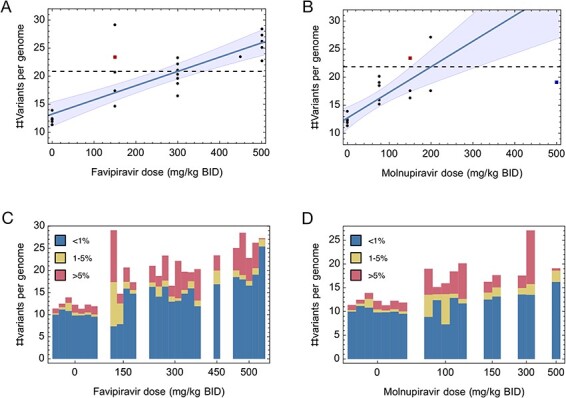
Statistics of virus diversity in treated and untreated populations. (A) Increases in the mean number of variants per viral genome were observed in populations treated with increasing doses of favipiravir. Black dots show genome-wide statistics calculated from populations: Each dot represents the viral population in a single hamster. The red square shows the mean value for populations treated with 300 mg favipiravir and 150 mg molnupiravir. The blue square at 500 mg molnupiravir shows a datapoint that was omitted from the regression calculation; this point is discussed in the main text. Shading indicates a 95 per cent confidence interval for the regression line. A horizontal dashed line indicates the model threshold at the point of effective treatment. (B) Increases in the mean number of variants per viral genome per virus were observed in populations treated with increasing doses of molnupiravir. (C) Composition of variants denoted by variant frequency, ordered by favipiraivir dose. Bars show the total number of variants per genome (plotted as points in Fig. 1A), decomposed into the contributions to this total from variants of different frequencies. Each bar describes statistics for the viral population in a single hamster. (D) Composition of variants denoted by variant frequency, ordered by molnupiravir dose. Bars show the total number of variants per genome (plotted as points in Fig. 1A), decomposed into the contributions to this total from variants of different frequencies.

Sequence data described the viral population in each hamster 4 days after the initiation of infection. Examining these data, a statistically significant relationship was identified between the dose of either molnupiravir or favipiravir and a measure of the diversity of the viral population at the time of sample collection. For each experimental population, we calculated the sum of all variant allele frequencies with respect to the consensus of the viral inoculum, irrespective of their frequency. We denote this statistic by *q*: it provides an estimate of the mean number of variants per virus. We found significant positive correlations between the dose of drug and the value of *q*; these correlations existed for both favipiravir and molnupiravir (*P*  < 2 × 10^–4^) (Fig. 1A, B). Our results suggest a link between the reduced fitness of the treated viral populations, seen in the reduced infectious viral titres associated with treatment, and the accumulation of mutations in the viral genomes. Our analysis suggests that molnupiravir may be more potent than favipiravir as a mutagenic agent, with a greater number of variants induced per microgram of drug, although this result did not achieve statistical significance (*P* = 0.063, Student’s *t*-test). Samples were collected following treatment with a combination of 150 mg/kg BID molnupiravir and 300 mg/kg BID favipiravir had a greater value of *q* than did populations treated with either drug individually (*P*-value 0.021 for molnupiravir, 0.011 for favipiravir). The increased sum of variants in this case suggests that that the two drugs may operate in a cooperative manner, with the addition of a second drug leading to a significant additional increase in the rate of viral mutation.

When evaluated in terms of the mean number of mutations per virus, the thresholds at which drug dosing became effective for SARS-CoV-2 treatment in the hamsters (i.e. 300 mg/kg BID for favipiriavir and 200 mg/kg BID for molnupiravir) (Kaptein et al. 2020; Abdelnabi et al. 2021a) were similar, with estimated values of *q* equal to 20.8 mutations per genome (relative to the initial consensus) for favipiravir, and 21.9 mutations per genome for molnupiravir in our regression model (Fig. 1A,B); these values represent a gain of between 7 and 10 variants per viral genome versus the untreated control, associated with effective drug treatment.

Our linear regression analysis excluded one data point, collected from a population treated with 500 mg/day BID of molnupiravir; our regression model would predict this to have a higher number of mutations per genome than were observed. A possible reason for this observation is that under a high dose of treatment viruses lost their potential for replication before a higher burden of mutations could accumulate. Alternatively, the higher dose of drug administered may not have led to a correspondingly higher dose in blood plasma (Nguyen et al. 2017). The collection of only a single data point at this dose prevented further investigation of this result.

Examining the frequencies of minor variants, we observed that the bulk of variation in viral populations was comprised of low-frequency variants, with, in most populations, more than half of the total variation consisting of variants at frequencies less than 1 per cent (Fig. 1C, D). A regression model showed a significant positive relationship between the drug dose and the sum of variants at frequencies of less than 1 per cent (Supplementary Fig. S1, *P* < 0.004). The preponderance of low-frequency mutations may reflect the combination of mutagenic treatment with a short period of infection, with limited time for de novo mutations to reach higher frequencies, even under positive selection (Morris et al. 2020). Sequencing error likely contributes to our overall sum of variant frequencies, and complex patterns of error have been identified in sequence data describing SARS-CoV-2 infection (Lythgoe et al. 2021). However, an analysis of sequence data found no correlation between read depth and the value of *q*, suggesting that our results were not purely artefactual (Supplementary Fig. S2).

**Figure 2.**
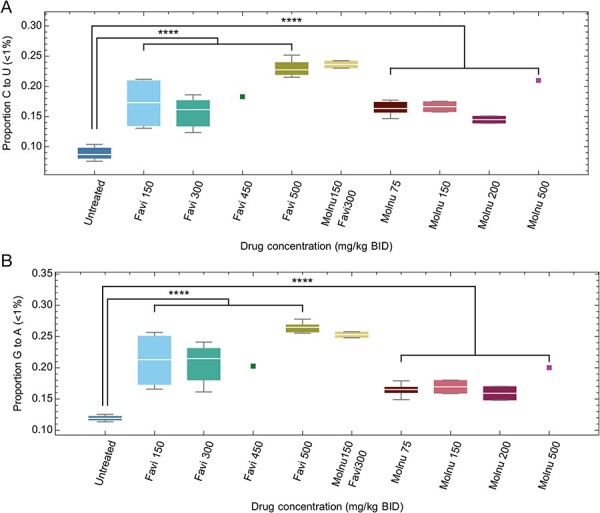
Mutational spectra of low-frequency variants. (A) The proportion of low-frequency variation (variant frequency <1 per cent) that was comprised of C to U mutations was higher for treated populations than for untreated populations. Squares show treatments for which a single data point was collected. (B) The proportion of low-frequency variation that was comprised of G to A mutations was higher for treated populations than for untreated populations.

In addition to changes in the amount of variation in viral populations, the use of favipiravir and molnupiravir were associated with significant change in the composition of genetic variants (Fig. 2). Examining the mutational spectra of variants with frequencies less than 1 per cent, we identified significantly higher proportions of C to T and G to A mutations in samples from treated, compared to untreated hamsters (*P* < 2 × 10^−6^, Student’s *t*-test). A previous analysis of the effect of favipiravir during long-term influenza B infection examined the same statistic for variants with frequencies of less than 5 per cent (Lumby et al. 2020); repeating the analysis with this higher frequency cut-off produced very similar results (Supplementary Fig. S3).

**Figure 3.**
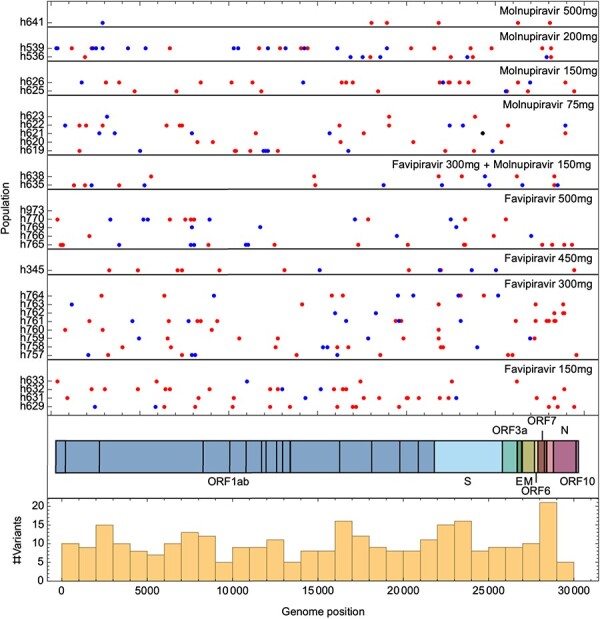
Genomic locations of variants in treated viral populations. The locations of variants which reached a frequency of at least 5 per cent in the viral population are shown in red (non-synonymous variant), blue (synonymous variant) or black (nonsense variant). With the exception of variants transmitted through standing variation, very little replication of variants between treated populations was observed.

**Figure 4.**
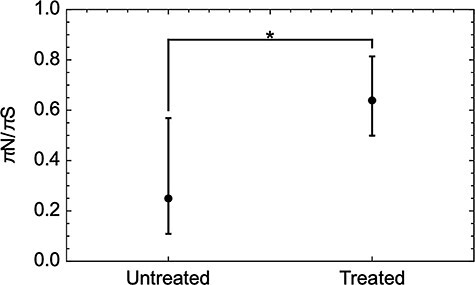
πN/πS was lower in untreated than in treated populations. Error bars were calculated using a likelihood model.

Considering variants at higher frequencies, we identified a total of 306 de novo variants at frequencies of 5 per cent or greater within the treated populations (Fig. 3). Of these variants, 207 were non-synonymous with 98 synonymous and one nonsense mutation. Accounting for the proportion of sites that could engender synonymous or non-synonymous mutations, these values indicate a bias towards synonymous variation, with a πN/πS ratio equal to 0.63. An equivalent calculation for variants in untreated populations gave significantly a lower πN/πS ratio of 0.25 (*P* < 0.02, likelihood ratio test, Fig. 4), indicating a greater bias against the emergence of nonsynonymous variants in untreated populations.

Among the variants identified at frequencies of 5 per cent or greater we did not identify a strong signal of repeated evolution, as might be expected if the virus was channelled into specific pathways of adaptation. Among the variants observed in the 30 treated populations, only 6 were observed in more than one of the populations. As a potential exception to this, the A21804G variant, which codes for the N81S mutation in the Spike protein, was observed as a consensus-level substitution (i.e. with a frequency in excess of 50 per cent) in two populations that were treated with favipiravir. Variants at frequencies 5 per cent or greater were roughly uniformly distributed across the genome, following a pattern seen in the general evolution of SARS-CoV-2 in the human population (Harari et al. 2022). In contrast to our identification of a link between the sum of variant allele frequencies and drug dose, there was no significant relationship between drug dose and the number of variants observed at frequencies of 5 per cent or greater (Supplementary Fig. S4), suggesting that the expansion of new variants to these frequencies was dominated by stochastic events.

**Figure 5.**
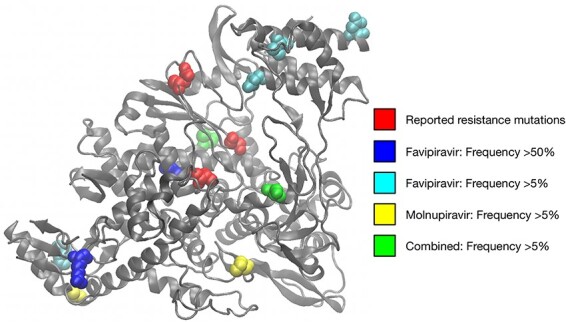
Variants observed in RdRp at frequencies of 5 per cent or greater. Sites reported to convey resistance to remdesivir are shown in red vdW representation (Szemiel et al. 2021; Gandhi et al. 2022; Stevens et al. 2022). Other colours show variants observed in hamster populations at frequencies of 5 per cent or more. Image created in VMD (Humphrey, Dalke, and Schulten 1996) based on the PDB structure 6m71 (Gao et al. 2020).

Among variants observed at frequencies of 5 per cent or greater, we did not find evidence of adaptation that could confer resistance against the antiviral drugs used in these experiments. The variants we observed did not replicate known remdesivir-resistance variants observed in SARS-CoV-2 (Szemiel et al. 2021; Gandhi et al. 2022; Stevens et al. 2022). Two consensus-level variants (i.e. > 50 per cent with respect to the original consensus) were observed in RdRp from hamsters given treatment with favipiravir, but nothing was observed at these frequencies following treatment with molnupiravir (Fig. 5). We did not identify mutations in the RdRp binding site, or in sites that have been associated with drug resistance for other RdRp inhibitors (Supplementary Table S1). Within the Spike protein, consensus-level variants were observed in populations treated with favipiravir, two of which coded for the N81S substitutin, while a single consensus-level variant was observed for populations treated with molnupiravir (Supplementary Fig. S5, Supplementary Table S2). One variant, I692V, observed at a frequency of 8 per cent in a population treated with molnupiravir, was among the set of variants which defined the B.1.1.198 variant of concern, identified early in the pandemic (Harvey et al. 2021). Although it is possible that treatment with mutagenic drugs could promote the emergence of new variants of concern, our data were not sufficient to draw firm conclusions on this hypothesis.

Taken together our results in combination suggest that the primary effect of short-term favipiravir and molnupiravir treatment upon SARS-CoV-2 genomes was an accumulation of low-frequency mutations, biased towards the accumulation of C to U and G to A variants, and without significant gain of variants at higher frequencies. Lower doses of antiviral drug, which did not have a significant effect upon viral load, had a smaller, but still evident, effect upon viral populations. The bias in our result towards the accumulation of low-frequency variants stands in contrast with cases that have described the extended use of mutagenic drugs. For example, a study of the prolonged treatment of norovirus infection with favipiravir showed a progressive accumulation of variants at higher frequencies (Ruis et al. 2018). It is likely that the shorter timespan of both infection and treatment in our experimental model underpin this difference.

## Discussion

Both molnupiravir and favipiravir are mutagenic antiviral drugs. When used against SARS-CoV-2 they interfere with viral replication, leading to an increase in the mutation rate of the virus. Increasing the mutation rate raises the possibility that consequent increased genetic variation will result in the evolution of drug resistance mutations, or of mutations that could make a virus better adapted to a new host. Previously, a SARS-CoV-2 infection model in Syrian hamsters showed a dose-dependent reduction in viral loads, infectivity, and lung pathology in molnupiravir- or favipiravir-treated animals (Kaptein et al. 2020; Abdelnabi et al. 2021a,b). Taking this further, we here used viral genome sequence data to explore how favipiravir and molnupiravir shaped the viral populations in these animals. Studying samples from hamsters provides an increased degree of replicability versus cases of human infection, and the short period of treatment (following initiation of infection with a large number of viruses) provides a reasonable model of the use of antivirals against a recently established case of human SARS-CoV-2 infection.

We identified statistically significant correlations between the dose of favipiravir or molnupiravir given to the animals and a statistic, calculated as the sum of the variant allele frequencies in the viral genome. The increase in our statistic was linked to an increase in low-frequency variation, comprising variants at <1 per cent frequency, which in most populations made up the majority of the total variation. Examination of the mutational spectra of populations treated with favipiravir and molnupiravir identified significant increases in the proportion of low-frequency variation that was comprised of C to U and G to A mutations.

Given the mutagenic action of favipiravir and molnupiravir, our result is not unexpected, but it provides a novel perspective upon how the use of mutagenic drugs could contribute to the global evolution of the virus. Our result is not unexpected in so far as an increase in the viral mutation rate would be expected to increase the extent of genetic variation in viral genomes. Given a short period of infection, there is limited time for novel mutations to reach high frequencies, so that most additional variation occurs at low frequency. Our result provides a novel perspective on the consequences of mutagenic drug use by means of our inclusion of low-frequency variation. A common procedure in the evaluation of viral sequence data is the identification of single-nucleotide polymorphisms, which on the basis of statistical analysis can be unambiguously identified in a population (Alteri et al. 2022; Donovan-Banfield et al. 2022). This identification usually requires the collection of a minimum number of reads, and for the variant to have been observed at a cut-off frequency, often of around 2 per cent (McCrone et al. 2018). While this approach identifies unambiguous individual variants, it potentially neglects changes in the viral population at lower frequencies. Although our statistic *q* will be affected by sequencing error, the consistent experimental procedures used in our experiment imply that differences between samples in that statistic should also be consistent.

Our estimate that effective treatment with favipiravir or molnupiravir led to between 7 and 10 additional variants per viral genome sequence provides some insight into the potential for mutagenic drugs to affect the global viral population. Transmission bottlenecks for SARS-CoV-2 are usually tight, with few viruses initiating new infections (Lythgoe et al. 2021; Bendall et al. 2023). Where a transmission bottleneck involves a single viral particle, any variants carried by the founder virus will be fixed in the recipient population. In this way, while selection could play a role in reducing the number of variants transmitted, and while treatment with mutagenic drugs will potentially reduce the mean viral load and hence the potential for transmission, the potential exists, if transmission does take place, for multiple variants to accumulate in the viral lineage in a short period of time. Long branches in the tree of SARS-CoV-2 sequences could potentially be created without the need for a long period of infection, or a need for variants to reach high frequencies in the donor individual.

Our study identified that an effective dose of treatment for both favipiravir and molnupiravir led to a viral population with close to 20 mutations per virus. Our statistic describing the number of variants per viral genome may have some utility as a clinical biomarker for evaluating the effectiveness of mutagenic drugs. However, several barriers remain to be overcome ahead of its use. First, a greater understanding would be required of how sequencing error affects the absolute value of this statistic: Different sequencing approaches may have distinct error profiles. Second, in our dataset identical doses of drug led to variable levels of viral load; further investigation of the extent of this variation would be required. Third, our samples were collected after four days. In the absence of multiple samples per individual we could not assess whether this statistic had reached an equilibrium value. Finally, hamsters likely represent a more homogeneous population than human patients. Even if a dose of drug were to produce a consistent fitness reduction, the intrinsic viral fecundity in the absence of treatment may vary according to the specific immune response of the individual and the genotype of the virus.

By contrast to our results for low-frequency variation, we did not identify a meaningful signal in the numbers of higher-frequency variants in populations treated with a higher drug dose. This result may also arise from the short time span of the experiment: Variants at higher frequencies take longer to emerge. The general lack of repeated variants in treated populations is consistent with a process whereby variants rose to high frequency by chance, via a random process of mutation and genetic drift. Against this background, we cannot exclude the possibility of having observed some genuinely beneficial mutations, whether conferring adaptation to the hamster host or some form of drug resistance. Adaptive viral evolution in multiple directions has previously been observed in experimental cases of animal infection (Illingworth 2015). Thus, a process in which distinct beneficial mutations emerged in different hosts cannot entirely be ruled out. Our identification of a clear and statistically significant signal in one metric of genetic variation, but the lack of a significant signal in another, highlights the need for care in using sequence data to evaluate the effects of mutagenic drugs on viral populations.

Our result concerning animals treated with suboptimal doses of drug validates suggestions that such treatment could increase viral genetic diversity without a significant reduction in viral load. A complex relationship likely exists between the potential for variant transmission and drug dose, with higher doses creating more variants in the viral population, but reducing the probability of transmission due to the reduction in viral load.

Despite their limitations, our results suggest that treatment with mutagenic drugs increased the numbers of mutations accumulating in SARS-CoV-2 in a roughly continuous manner, with the increase in load being proportional to the level of antiviral dosing. To the extent that mutagenic drugs result in a loss of viral infectivity, it can be concluded that the burden of mutations they impose upon the virus is deleterious, with successive gains of mutations being to the detriment of the viral population. On this basis, we would expect that, while suboptimal dosing could lead to the evolution of more mutated viruses, the emerged viruses would mostly be of reduced fitness compared to those transmitted from an untreated population. Our study did not find evidence for either the emergence of antiviral resistance, or the systematic emergence of beneficial variants that would militate against the clinical use of mutagenic drugs. In the absence of specific evidence of negative effects, clinical studies describing the efficacy (or otherwise) of treatment should be given priority in determining the use of mutagenic antiviral drugs.

## Methods

### Virus samples

The data for this study was generated from a Syrian hamster model designed to demonstrate the efficacy of short term (4 days) molnupiravir and favipiravir in reducing SARS-CoV-2 viral load, infectivity and lung pathology. Details of these experiments have been described in previous publications (Kaptein et al. 2020; Abdelnabi et al. 2021a,b). Large numbers of viral genomes were recovered from these animals (Supplementary Fig. S6).

### Sequence analysis

Fastq files were processed with Trimmomatic (Bolger, Lohse, and Usadel 2014) using default settings, retaining reads for which both reads in a pair passed filtering. The remaining reads were aligned to the MN908947.3 reference sequence using bwa (Li and Durbin 2009), before using samtools (Li et al. 2009) to remove reads that did not align to the primary alignment. Further processing was conducted using the SAMFIRE package (Illingworth 2016). Filtering was conducted to remove low quality base pairs (PHRED score <30), following which variants were identified genome-wide. Samples were kept for analysis if the mean coverage of the viral genome was at least 150×. Applying this criteria gave us samples from a total of 7 untreated hamsters, 17 hamsters treated with favipiravir (3 at 150 mg, 8 at 300 mg, 1 at 450 mg, and 5 at 500 mg), and 10 hamsters treated with molnupiravir (5 at 75 mg, 2 at 150 mg, 2 at 200 mg 1 at 500 mg), alongside 2 hamsters treated with both favipiravir and molnupiravir. A full list of samples is provided in Supplementary Table S3.

### Number of variants per genome

An estimate of the mean number of variants per viral genome was calculated using the aligned sequence data. We assessed this statistic by calculating the sum of allele frequencies across the genome. We first identified consensus alleles as the most common nucleotides present in the inoculum population. At position i in the genome we then calculated the frequency q_ia_ of the allele a for each non-consensus allele a, considering sites with a coverage of at least 150 reads but with no filter on variant allele frequencies. We then calculated the sum


$$q = \mathop \sum \limits_i \mathop \sum \limits_a {q_{ia}}$$

The sum in this case encompasses all variants, irrespective of their frequency. In this calculation, requiring 150 reads at a site removes the potential for sites with low-read depth and a small number of erroneous reads to unduly bias our measurement of *q*, but comes at the cost of potentially excluding genuine variation at poorly covered sites. Reducing the threshold number of reads required to 50 or 10, or removing this requirement altogether did not produce dramatic changes in the statistics obtained (Supplementary Fig. S7), with extremely strong linear relationships identified between values obtained under different thresholds.

Our use of linear regression to analyse these data was motivated by a crude model of variant evolution at low frequencies. Making the assumption that a site in a genome may contain only two possible alleles, and ignoring linkage between variants, the frequency of a deleterious allele is expected to tend, under mutation-selection balance, to an equilibrium frequency of *x* = *μ/s*, where *μ* is the mutation rate and *s* is the magnitude of selection against the allele (Haldane 1937), according to the equation


$$\frac{{dx}}{{dt}} = \mu - sx$$

Furthermore, assuming mutation rates from one allele to the other are equal, the frequency of a neutral allele is expected to tend to a frequency of one half, according to the equation


$$\frac{{dx}}{{dt}} = \mu \left( {1 - 2x} \right)$$

In both of these formulas, the change in the frequency x is proportional to μ when x is small. Although positive selection and linkage disequilibrium will affect the evolution of the viral population, we nevertheless sought to fit a linear model to our data.

### Analysis of variant composition

The proportion of low-frequency variation of distinct mutational classes was measured using variants at frequencies of 1 per cent or below. Briefly, the calculation of q was repeated, considering exclusively low-frequency variants, following which the proportion of this sum that is comprised of each of the 12 mutational classes was calculated.

### Non-synonymous and synonymous variation

In order to explore the potential adaptive evolution of the viral populations, we identified variants which had reached a frequency of 5 per cent or more. To analyse the composition of these variants, we calculated *πN*/*πS*, defined as


$$\frac{{\pi N}}{{\pi S}} = \frac{{cN/oN}}{{cS/oS}}$$

Where *cN* and *cS* were the genome-wide counts of non-synonymous and synonymous variants reaching a frequency of 5 per cent or more, and *oN* and *oS* are the number of potential non-synonymous and synonymous variants that could arise given the various reading frames in the SARS-CoV-2 genome, using the consensus sequence of the initial viral population as a reference. A value of this statistic less than one suggests that the emergence of variants to a frequency of 5 per cent or greater was biased by selection against nonsynonymous variants.

To calculate a *P*-value for the difference between *πN*/*πS* values, we calculated likelihoods for the underlying proportions *p_U_* and *p_T_* of variants in the untreated and treated populations which were non-synonymous, relative to the opportunity, and based upon the observations made. Where we observed *cN_U_* nonsynonymous variants and *cS_U_* synonymous variants in the untreated population, and *cN_T_* nonsynonymous variants and *cS_T_* synonymous variants in the treated population, a likelihood for the values *p_U_* and *p_T_* is given by


$$L\left( {{p_U},{p_T}} \right) & = \left( {\begin{array}{*{20}{c}}
{c{N_U} + c{S_U}}\\
{c{N_U}}
\end{array}} \right){\left( {{p_U}} \right)^{c{N_U}}}{\left( {1 - {p_U}} \right)^{c{S_U}}}\left( {\begin{array}{*{20}{c}}
{c{N_T} + c{S_T}}\\
{c{N_T}}
\end{array}} \right) \\ & {\left( {{p_T}} \right)^{c{N_T}}}{\left( {1 - {p_T}} \right)^{c{S_T}}}$$

This distribution is shown in Supplementary Fig. S8. Defining the hypothesis H_1_: *p_U_* < *p_T_*, and H_0_: *p_U_* ≥ *p_T_*, and integrating over the corresponding regions of this distribution, we obtained the p-value given in the main text.

### Identification of spike mutations of interest

As the hamsters were infected with Wuhan-like virus, we assumed mutations of interest would be those identified in the human population in variants of concern/variants of interest. Mutations arising were looked up in tables of mutations with antibody escape information from a previous publication and referenced material (Harvey et al. 2021).

### Visualization of protein structures

Images of protein structures were made with the Visual Molecular Dynamics software package (Humphrey, Dalke, and Schulten 1996).

## Supplementary Material

veae001_Supp

## Data Availability

Sequence data are available from the Sequence Read Archive with accession number PRJNA935666.
