# Supplementary figures and images for: Genetic consequences of effective and suboptimal dosing with mutagenic drugs in a hamster model of SARS-CoV-2 infection
Source: Virus Evol. 2024 Jan 4;10(1):veae001. doi: 10.1093/ve/veae001 (PMC10939363; doi:10.1093/ve/veae001)

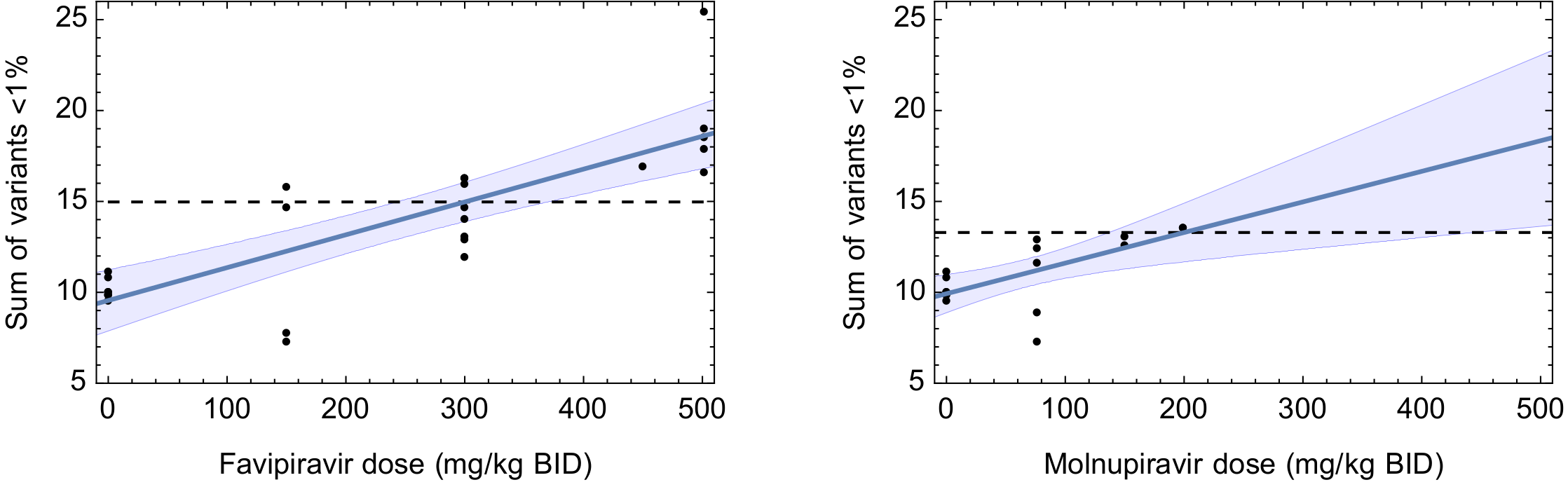

Supplement: veae001_Supp [file veae001_supp.zip › suppl_data/FigureS1.tif]

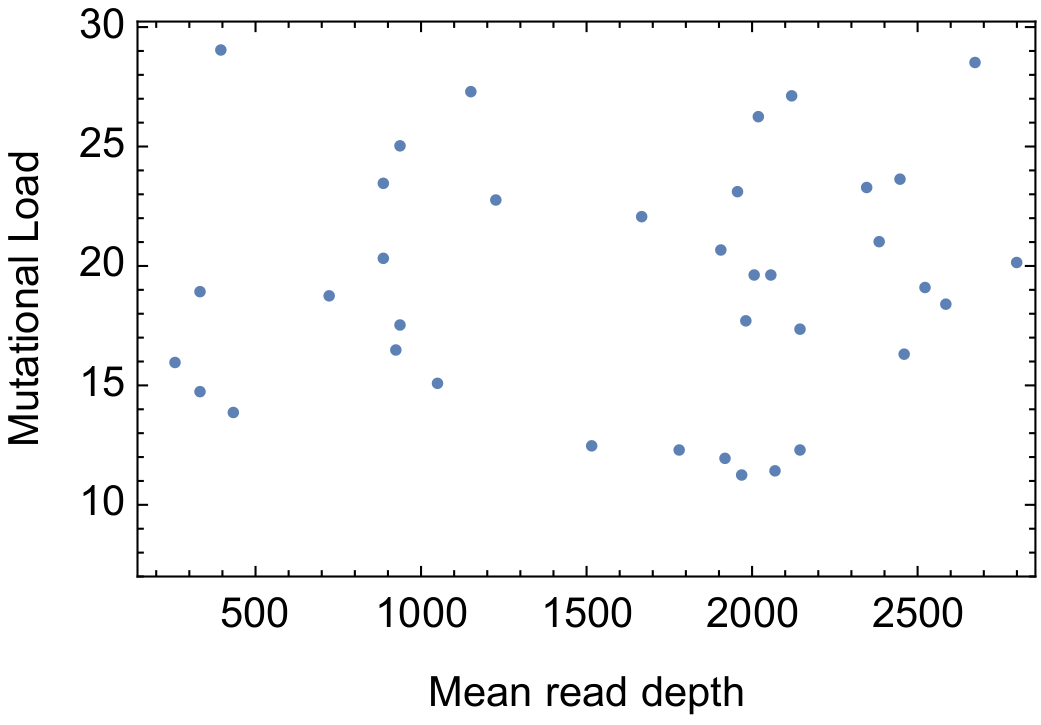

Supplement: veae001_Supp [file veae001_supp.zip › suppl_data/FigureS2.tif]

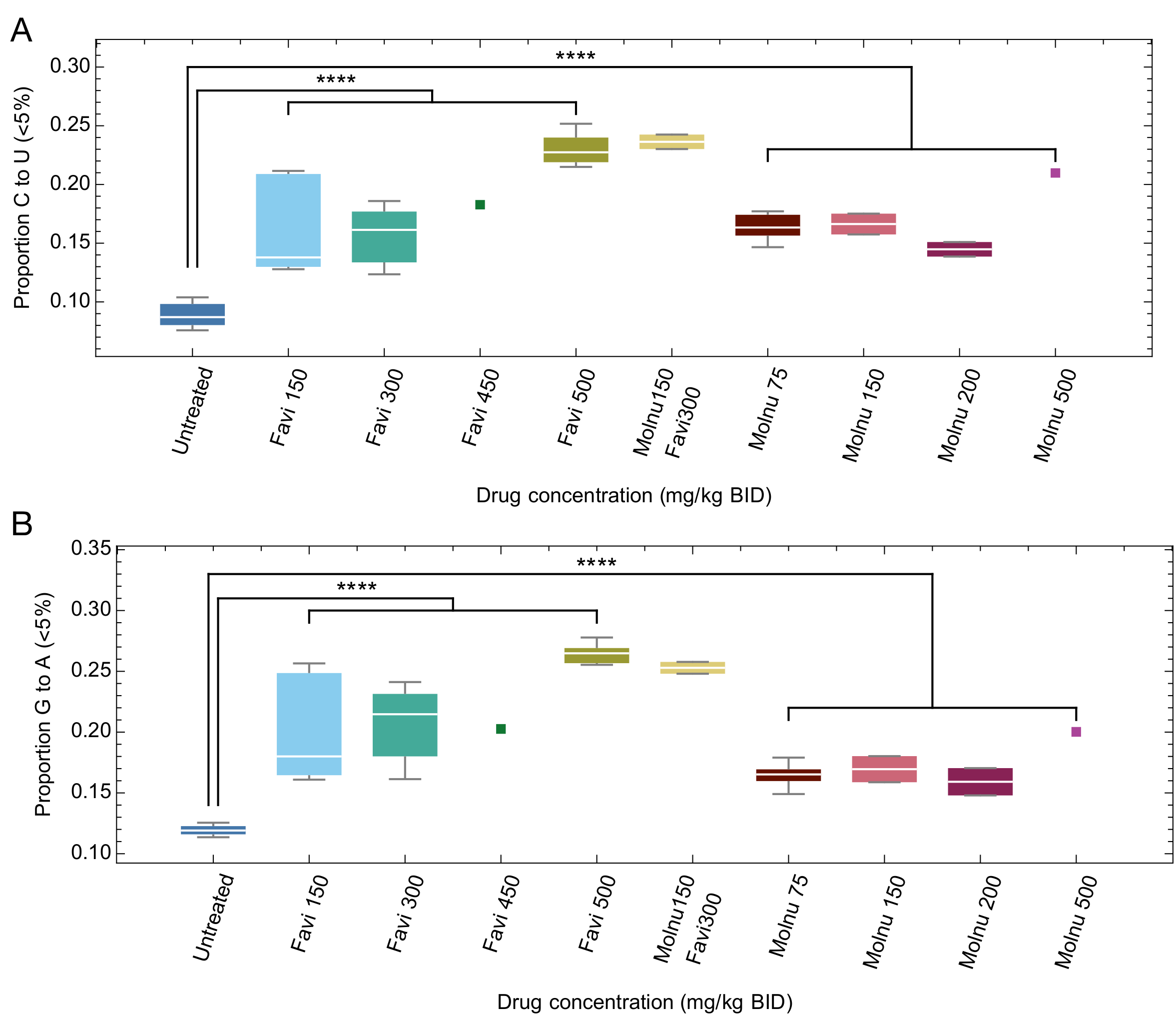

Supplement: veae001_Supp [file veae001_supp.zip › suppl_data/FigureS3.tif]

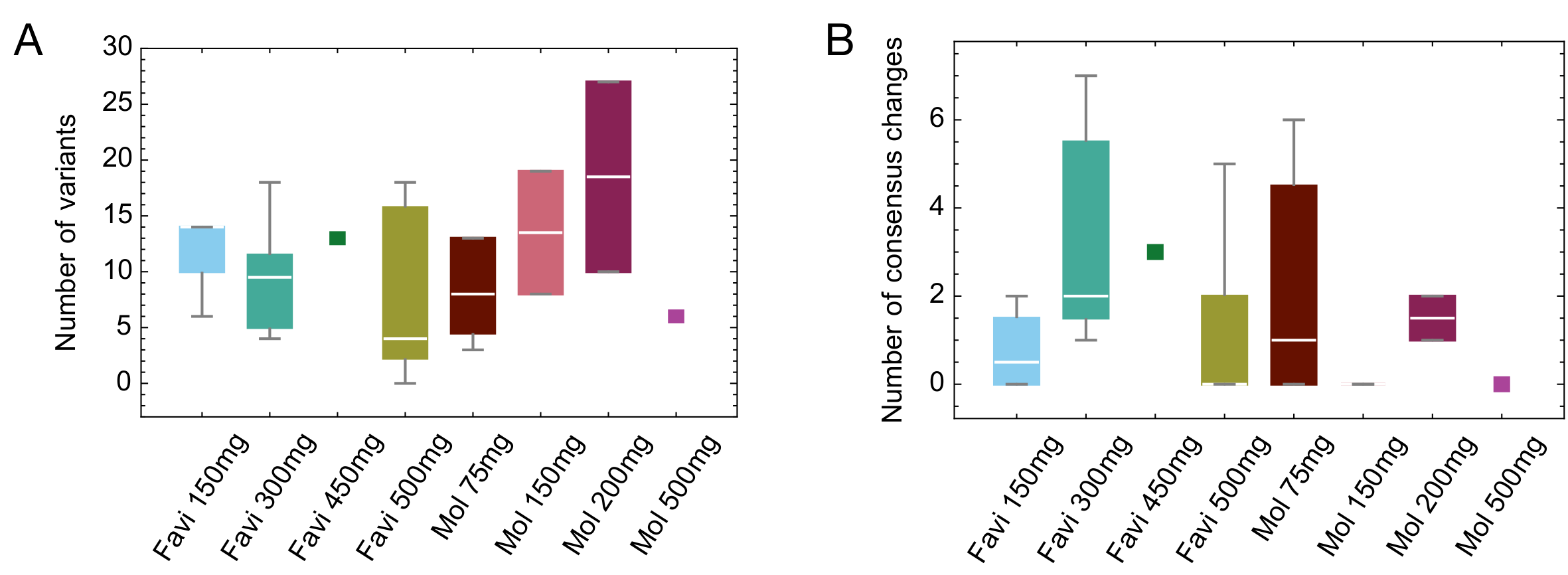

Supplement: veae001_Supp [file veae001_supp.zip › suppl_data/FigureS4.tif]

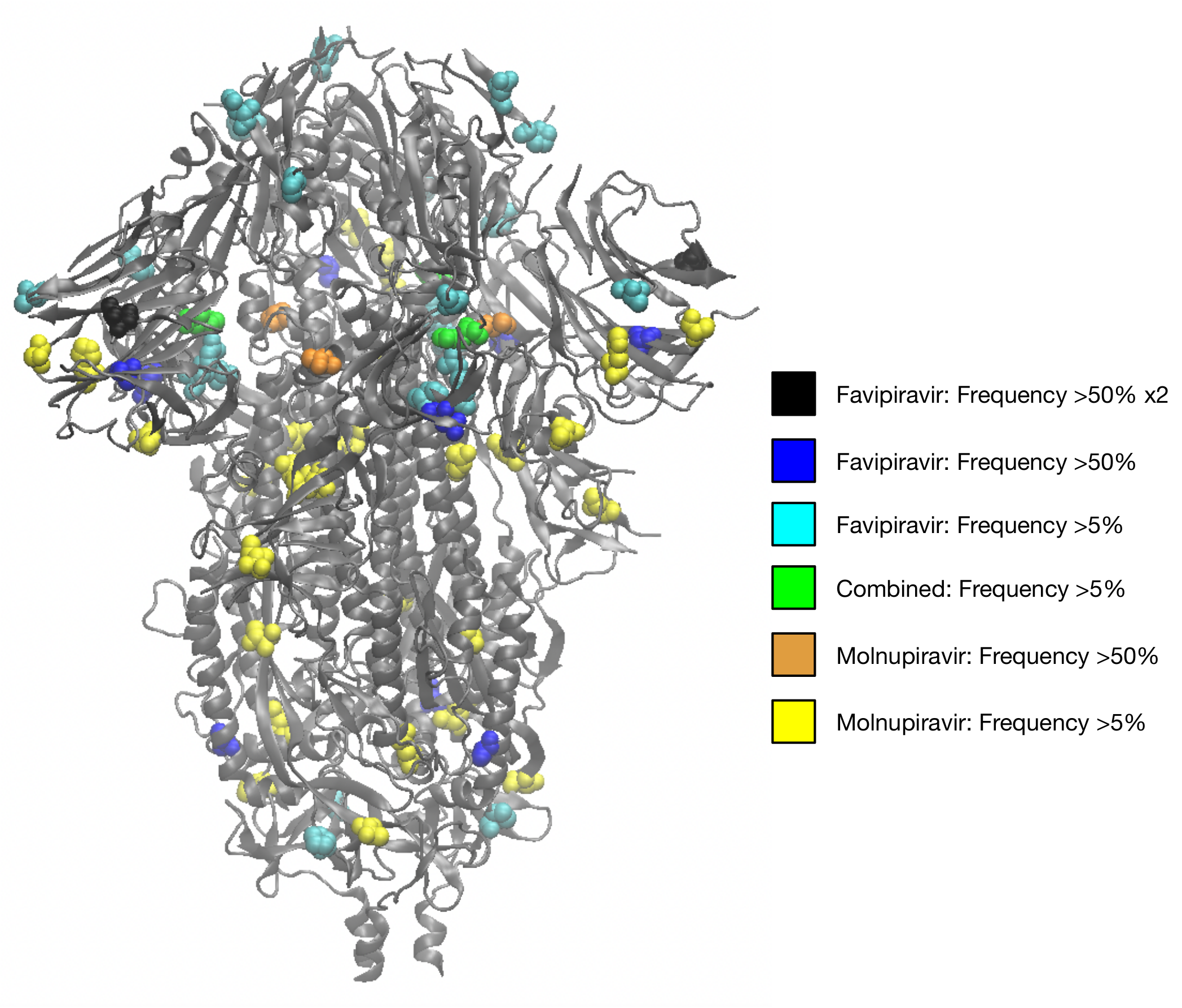

Supplement: veae001_Supp [file veae001_supp.zip › suppl_data/FigureS5.tif]

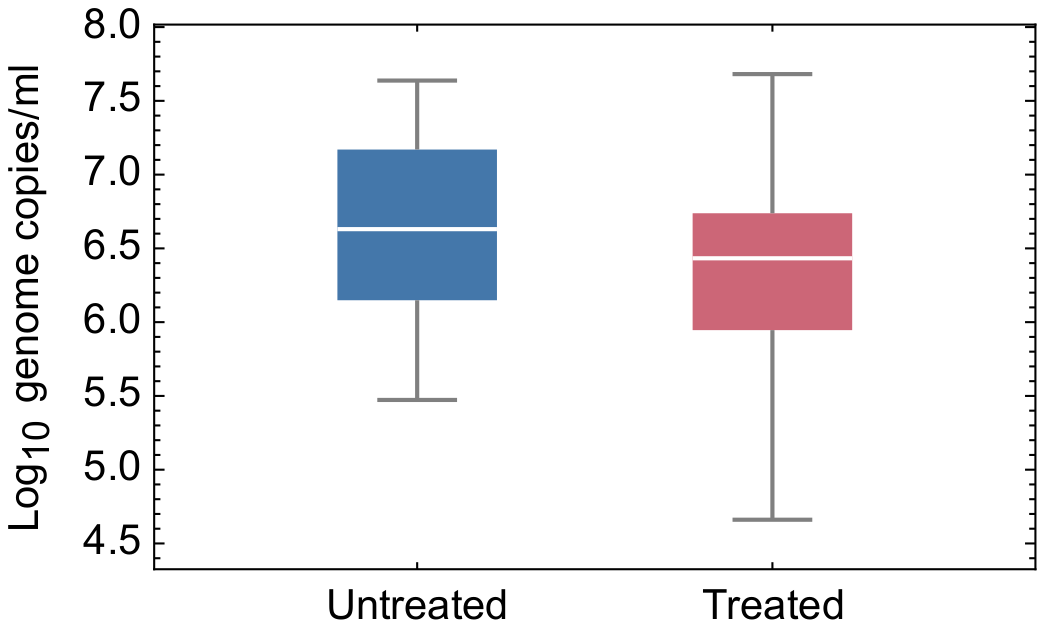

Supplement: veae001_Supp [file veae001_supp.zip › suppl_data/FigureS6.tif]

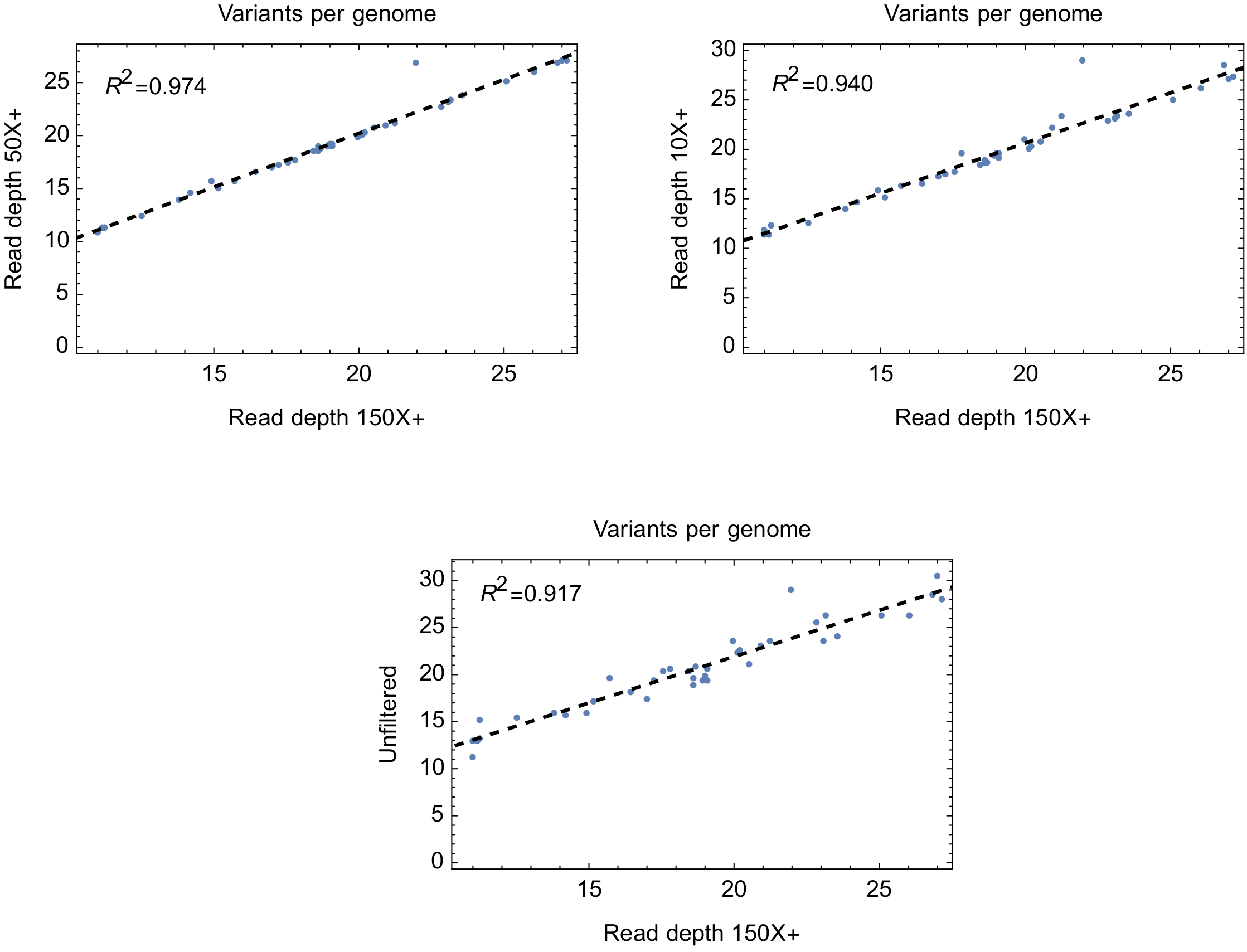

Supplement: veae001_Supp [file veae001_supp.zip › suppl_data/FigureS7.tif]

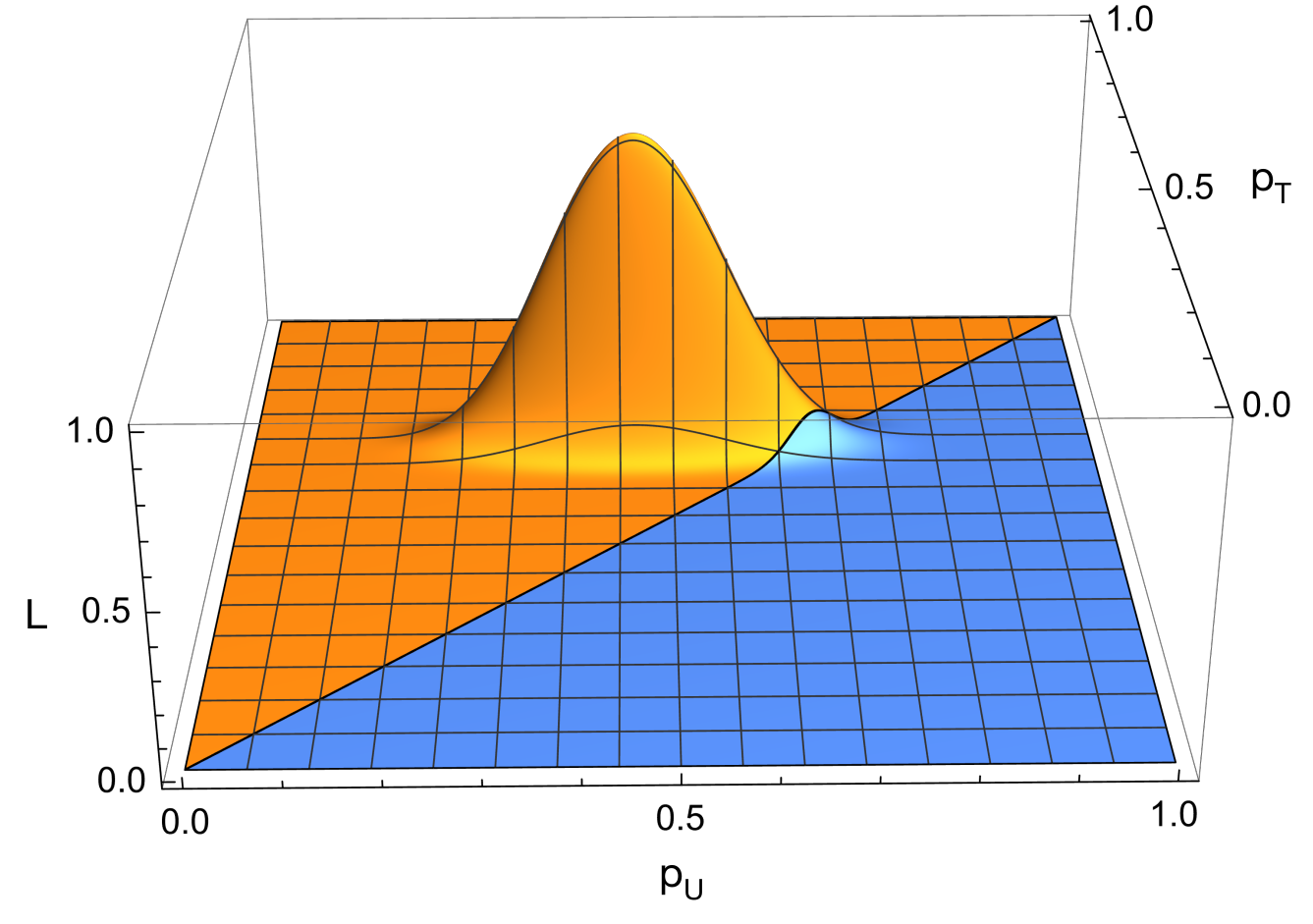

Supplement: veae001_Supp [file veae001_supp.zip › suppl_data/FigureS8.tif]
